# Acute Necrotizing Encephalopathy of Childhood: A Multicenter Experience in Saudi Arabia

**DOI:** 10.3389/fped.2020.00526

**Published:** 2020-10-09

**Authors:** Fahad A. Bashiri, Sultan Al Johani, Muddathir H. Hamad, Amal Y. Kentab, Ali H. Alwadei, Khalid Hundallah, Hamdi H. Hasan, Walaa Alshuaibi, Lamyaa Jad, Muhammad Talal Alrifai, Abrar Hudairi, Rana Al Sheikh, Asma'a Alenizi, Nawaf A. Alharthi, Tayseer A. Abdelmagid, Duaa Ba-Armah, Mustafa A. Salih, Brahim Tabarki

**Affiliations:** ^1^Division of Pediatric Neurology, Department of Pediatrics, College of Medicine, King Saud University, Riyadh, Saudi Arabia; ^2^Pediatric Neurology Department, National Neuroscience Institute, King Fahd Medical City, Riyadh, Saudi Arabia; ^3^Division of Pediatric Neurology, Department of Pediatrics, Prince Sultan Military Medical City, Riyadh, Saudi Arabia; ^4^Neuroradiology Division, Department of Radiology, College of Medicine, King Saud University, Riyadh, Saudi Arabia; ^5^Medical Genetics Division, Department of Pediatrics, King Khalid University Hospital, College of Medicine, King Saud University, Riyadh, Saudi Arabia; ^6^Division of Neurology, Pediatric Department, King Abdullah Children Hospital, King Abdulaziz Medical City, Ministry of National Guard, Riyadh, Saudi Arabia; ^7^Pediatric Department, King Abdullah bin Abdulaziz University Hospital, College of Medicine, Princess Nourah Bint Abdulrahman University, Riyadh, Saudi Arabia; ^8^Department of Pediatrics, Armed Forces Hospital Southern Region, Khamis Mushayt, Saudi Arabia

**Keywords:** necrotizing encephalopathy, children, seizure, influenza virus, RANBP2 gene

## Abstract

**Background:** Acute necrotizing encephalopathy of childhood (ANEC) is a rapidly progressing encephalopathy characterized by fever, depressed level of consciousness, and seizures. Diagnosis depends on clinical presentation and characteristic neuroimaging findings of abnormal signal intensity involving the thalami as well as the supra and infra-tentorial areas. Treatment modalities are not well-established; empirical treatment with antibiotics and antiviral agents is the initial step, followed by steroids and immunoglobulin, as well as supportive care. Patients with ANEC have a variable prognosis, but mortality is very high.

**Methods:** A retrospective chart review of patients diagnosed with ANEC in five tertiary centers from January 2015 to October 2018 was performed. Clinical and radiological findings, as well as the therapeutic approach and outcomes, were described.

**Results:** Twelve children were included ranging in age from 10 months to 6 years. All patients presented with preceding febrile illness, altered level of consciousness, and seizure. Radiological features showed abnormal signals in the thalami, and five patients (41.7%) had brainstem involvement. All patients received empirical treatment with antibiotics and antiviral agents. Ten patients (83.3%) received intravenous immunoglobulin (IVIG) and IV Methylprednisolone therapy. Outcomes were variable ranging from good outcomes with minimal neurological deficits to poor outcomes and death in 25% of cases.

**Conclusion:** ANEC is a rare fulminant disease in children. The treatment is challenging. Early interventions with the use of IVIG and IV Methylprednisolone may change the outcome; however, further studies are needed to establish a consensus guideline for the management.

## Introduction

Acute necrotizing encephalopathy of childhood (ANEC) is a fulminant type of encephalopathy. Most reported cases occur in Asian children with the highest prevalence among patients between the age of 6 and 18 months (1). The most common clinical presentations are fever, rapid alteration in the level of consciousness, and seizures, in addition to characteristic findings in brain imaging that include, but are not limited to, bilateral thalamic lesions with supra and infra-tentorial lesions of variable dimensions (2). The diagnosis of ANEC was determined by specific diagnostic criteria as described by Mizuguchi (1) which consist of (1) encephalopathy preceded by viral febrile illness with rapid deterioration in the level of consciousness and convulsions. (2) Absent cerebrospinal fluid (CSF) pleocytosis. (3) Symmetric multifocal brain lesions. (4) Elevation in serum aminotransferase levels. (5) Exclusion of similar diseases. The most frequent etiologies are viral-related, but sporadic cases of non-viral ANEC have been reported (3). Influenza virus has an important role in causing ANEC. In 1994, it was noticed that the number of cases of ANEC in Japan increased after discontinuation of the influenza vaccine that year (4). It was observed previously that influenza-associated ANEC is characterized by an increase in serum cytokine concentrations (interleukin-6 and interleukin-10) as compared to controls (5). The association between *RANBP2* gene pathogenic variants and susceptibility to ANEC has been addressed in previous studies (6–8). In this study, we aimed to present the clinical and radiological features, as well as treatment modalities and outcomes of 12 children with ANEC following viral infection in five tertiary care centers in Saudi Arabia.

## Methods

This was a retrospective review of patients who were diagnosed with ANEC in five tertiary centers in the Kingdom of Saudi Arabia from January 2015 to October 2018. The diagnosis of ANEC was determined by specific characteristic findings proposed by Mizuguchi (1) and Mizuguchi et al. (2). We included patients who were under 18 years and presented with clinical features consistent with ANEC in the form of altered level of consciousness, and seizures, preceded by viral illness with typical brain magnetic resonance imaging (MRI) findings of ANEC which include symmetrical, multifocal brain lesions involving mainly both thalami. Clinical characteristics and radiological features, as well as laboratory investigations, were collected. Other differential diagnoses were excluded based on clinical and radiological features as well as metabolic screening. All patients had normal serum ammonia, normal basic metabolic workup, including serum lactate, amino acids, and urine for organic acids. We described the outcome based on the follow-up visits in neurology clinics. The need to obtain informed consent was waived for a retrospective review of patient records.

### Statistical Analysis

The mean standard deviation was used to describe continuous variables, categorically measured variables were described using frequency and percentage. The Pearson's correlation test (r) was used to assess the correlation between continuous variables and the Fischer's exact test and Chi Square tests were used for assessing the correlation between categorically measured variables. The multiple response dichotomies analysis was employed to assess the combined incidence of various signs and symptoms and presenting complaints of ANEC diagnosed patients at admission time due to the possibility of presenting with different combinations of symptoms and signs.

The study was approved by the institutional review board and ethical committee of the College of Medicine, King Saud University, Riyadh, Kingdom of Saudi Arabia (KSU-IRB Number 18/0683).

## Results

### Clinical Presentation and Radiological Features

**Figure 1:**
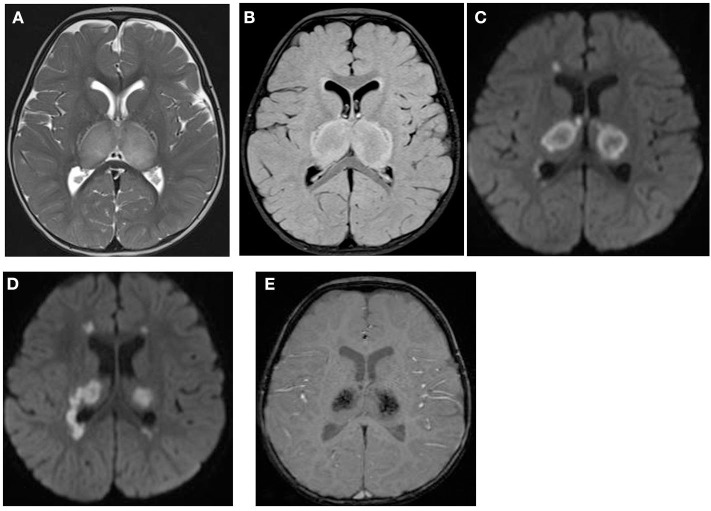
Brain MRI. Axial T2 **(A)**, FLAIR **(B)**, diffusion **(C,D)**, and susceptibility **(E)** weighted images. There are swelling and abnormal high signal intensity of both thalami **(A,B)** with diffusion restriction **(C,D)** and dark signal intensity with blooming **(E)**.

**Figure 2:**
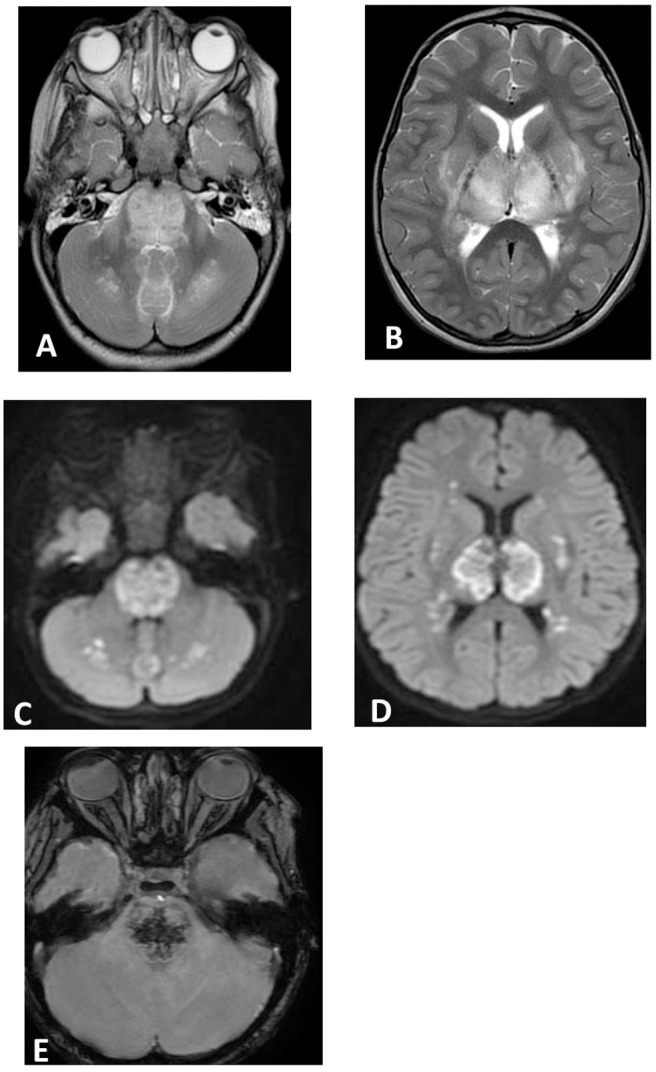
Brain MRI. Axial T2 **(A,B)**, diffusion **(C,D)**, and susceptibility **(E)** weighted images. There are swelling and abnormal high signal intensity of pons, bilateral cerebellar white matter **(A)**, both thalami, and bilateral putamen **(B)** with diffusion restriction **(C,D)** and dark signal intensity with blooming in the pons **(E)**.

**Figure 3:**
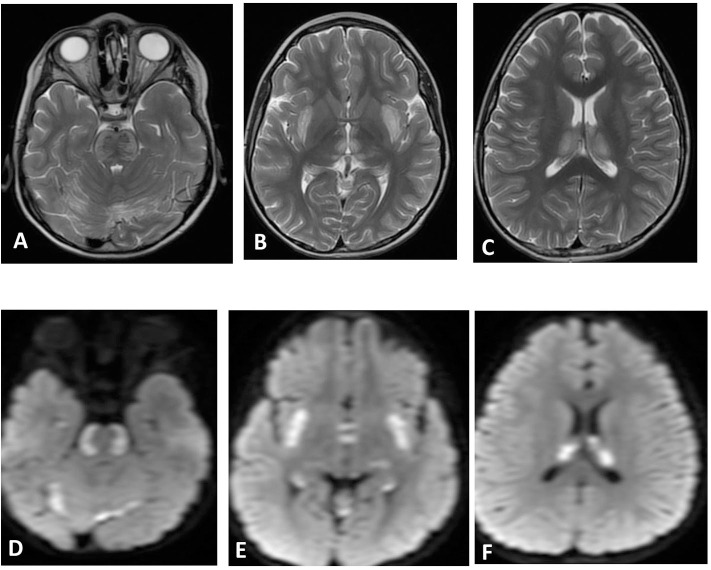
Brain MRI. Axial T2 **(A–C)** and diffusion **(D–F)** showing swelling and abnormal high signal intensity of pons, bilateral external capsule, and subinsular cortex, as well as both thalami with diffusion restriction.

**Figure 4:**
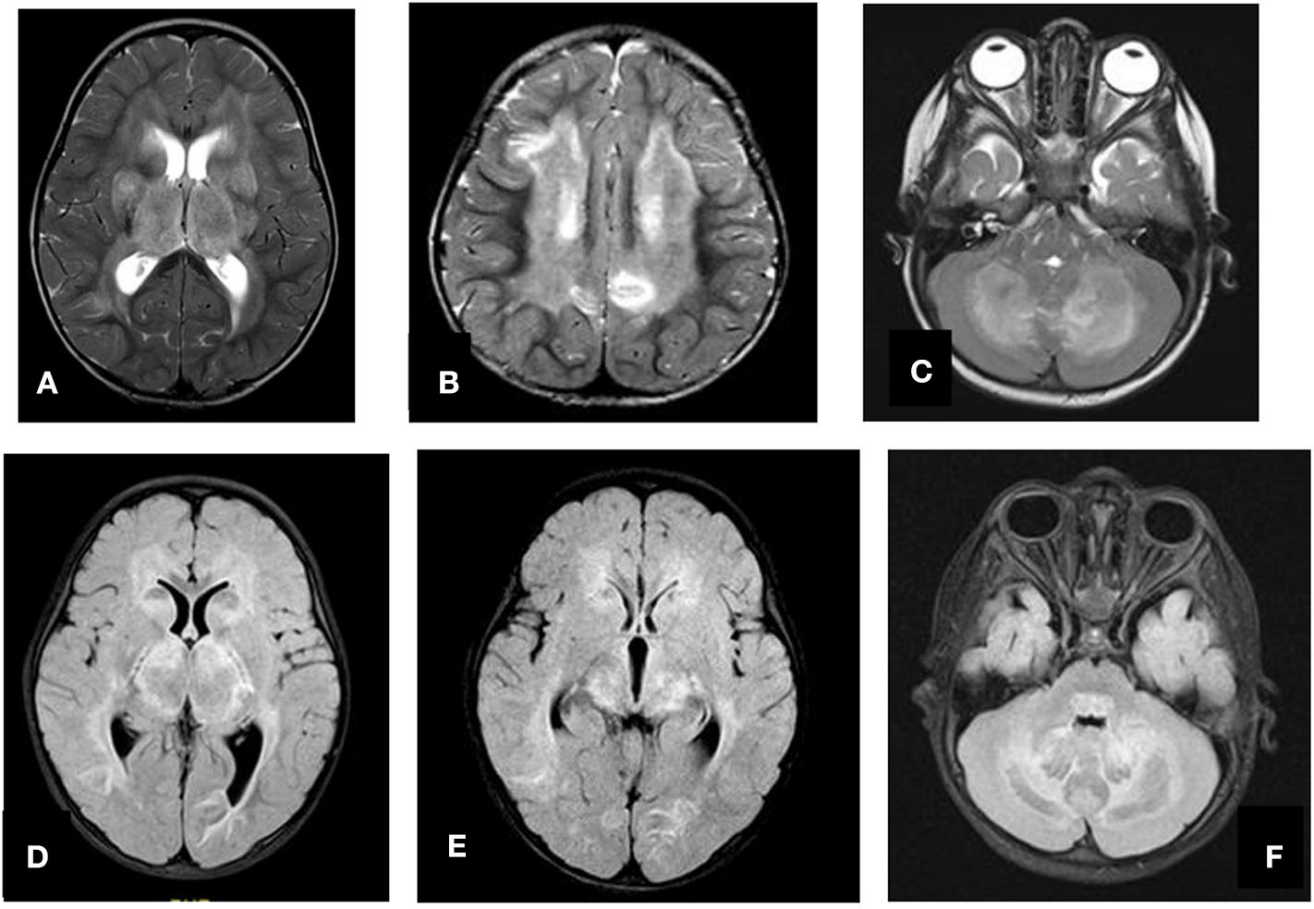
Axial T2 **(A–C)** and FLAIR **(D–F)** showing swelling and abnormal high signal intensity of both thalami, basal ganglia, and cerebellum.

A total of 14 patients with ANEC were identified; two patients were excluded due to the inability to meet the ANEC radiological criteria. Patients' ages ranged from 10 months to 6 years (mean 30.92 months, median 22 months, standard deviation 20.63). Nine patients (75%) were female. All patients presented with preceding febrile illness in the form of upper respiratory tract infection (URTI) symptoms, or gastrointestinal symptoms in the form of vomiting or diarrhea, and altered level of consciousness. Seizures were the initial presentation in eight cases (66.7%). Brain imaging was carried out using Axial, Sagittal, and Coronal T1,T2, FLAIR, diffusion, and susceptibility weighted images which showed characteristic high signal intensity on axial T2 and FLAIR with variable degree of involvement of both thalami in all cases. Diffusion restriction was also seen, while the susceptibility sequence showed dark signal intensity with blooming, indicating hemorrhagic changes. Out of 12 patients, five (41.7%) had brainstem involvement. Follow up brain MRIs were done 2–3 months after the initial presentation, which showed signs of improvement on 33.3%, and 66.7% (*n* = 8) showed no signs of improvement. The clinical and radiological presentations are summarized in Tables 1, 2. Figures 1–4 show brain MRIs for selected patients.

**Table 1:** Clinical characteristics and radiological features of the patients (*n* = 12).

**Case**	**Age/Gender Preceding illness**	**Initial presentation**	**Initial brain imaging lesions**	**Follow-up brain MRI**
1	17 Mo/F Fever/URTI/Chest infection	Fever/Seizures	MRI: bilateral thalami, basal ganglia, cerebellum	Resolution of previously noted lesions with small hemorrhagic foci; residual periventricular cystic lesions noted
2	2 Y/F Fever/URTI/Chest infection	Fever/Seizures	MRI: bilateral thalami, basal ganglia, brainstem (mainly pons)	Bilateral thalamic necrotic areas, with lacunar infarctions in periventricular and cerebellar white matter; global brain atrophy
3	5 Y/M Fever/URTI	Fever/Seizures	MRI: bilateral thalami, periventricular	Reduction of the intensity of the lesions with necrotic areas
4	16 Mo/M Fever/URTI	Fever/Seizures/Decreased consciousness	MRI: bilateral thalami, brainstem (mainly pons)	Not done
5	23 Mo/F Fever/Vomiting/Diarrhea	Fever/Seizures/Decreased consciousness	MRI: Bilateral thalami, cerebral white matter	Not done
6	6 Y/M Fever/Headache/Vomiting	Fever/Decreased consciousness	MRI: bilateral thalami, external capsule, brainstem, cerebellum	Reduction of the intensity of the lesions with necrotic areas
7	Fever/Seizures	26 Mo/F Fever/URTI	MRI: diffuse bilateral thalami, cerebrum, cerebellum, basal ganglia, brainstem	Not done (patient deceased within 24 h)
8	22 Mo/F Fever/URTI	Fever/Seizures/Decreased consciousness	CT: bilateral thalami, left basal ganglia, bilateral frontal lobes, diffuse brain edema	Not done (patient deceased after 48 h)
9	10 Mo/F Fever	Fever/Decreased consciousness	MRI: bilateral thalami, cerebellum, brain stem	Not done (patient deceased 1 month later)
10	5 Y/F Fever/Vomiting	Fever/Ataxic gait/Decreased consciousness	MRI: bilateral thalami, pons	Interval regression of bilateral thalami involvement
11	2 Y/F Fever/Diarrhea	Fever/Decreased consciousness	MRI: bilateral thalami, cerebellum, brain stem	Interval regression of bilateral thalami involvement
12	17 Mo/F Fever/URTI	Fever/Seizures/Decreased consciousness	MRI: Bilateral thalami, basal ganglia, internal capsule, pontine tegmentum, cerebellum, centrum semiovale, frontoparietal subcortical white matter, associated with diffusion restriction.	Interval regression of the lesions and development of hemorrhagic changes in both thalami, caudate heads, pontine tegmentum and cerebellum. Interval regression of the diffusion restriction. Mild dilatation of the ventricles.

*Mo, Months; Y, Year; F, Female; M, Male; CT, computed tomography; MRI, magnetic resonance imaging; URTI, upper respiratory tract infection*.

**Table 2:** Clinical characteristics and radiological features of the patients.

	**Frequency (*n* = 12)**	**Percentage (%)**
**Preceding symptoms/complaints**		
Fever	12	100
Upper Respiratory Tract Infection-URTI	7	58.3
Chest infection	2	16.7
Diarrhea	2	16.7
Vomiting	3	25
Headaches	1	8.3
**Presenting illness symptoms and signs**		
Fever	12	100
Seizure	8	66.7
Decreased sensorium/consciousness	8	66.7
Ataxic gait	1	8.3
**Brain MRI Initial findings/lesion locations (patients had multiple lesions)**		
Cerebrum	2	16.7
Basal ganglia	6	50
Cerebellum	6	50
Brain stem	5	41.7
Periventricular	1	8.3
Cerebral white matter	1	8.3
Internal capsule	1	8.3
Pons	4	33.3
Centrum semiovale	1	8.3
Bilateral frontal lobes	1	8.3
External capsule	1	8.3
Bilateral thalamus	12	100
**Follow up brain MRI at 6 months**		
Improved	4	33.3
Not improved	8	66.7

### Laboratory Investigations

All patients had a preceding viral illness. Nasopharyngeal aspirate (NPA) was positive for influenza A in 6 (50% of patients), whereas one patient had adenovirus, and the remaining (5 patients) had no isolated viral agents. A CSF polymerase chain reaction test for influenza A, enteroviruses and Herpes simplex virus was negative in the 10 tested patients.

Patients showed variable complete blood count (CBC) findings. Five patients (41.7%) had mild reversible thrombocytopenia and three developed leukocytosis. High levels of liver enzymes on initial presentation were detected in 66.7% of patients. All patients had a normal serum ammonia level. Basic metabolic workup, including serum amino acid, urine for organic acid, and serum lactate, were normal. CSF testing was done for all patients except two of them due to hemodynamic instability. Patients who underwent CSF testing had a normal cell count. Bacteriology culture studies were negative, Influenza virus and other viruses such as herpes simplex virus and human herpesvirus-6 were not isolated in the CSF, including patients with a positive nasopharyngeal aspiration (NPA) test for influenza A. CSF glucose and protein levels were variable, ranging from 3.2 to 5.6 mmol/L and from 0.19 to 1.65 g/dL, respectively. Laboratory findings are summarized in Table 3.

**Table 3:** Laboratory investigations.

	**Frequency (*n* = 12)**	**Percentage (%)**
**Complete blood count findings**		
Complete blood count (normal)	3	25
Thrombocytopenia	5	41.7
Anemia	1	8.3
Leukocytosis	3	25
**Liver function test findings**		
AST		
High	8	66.7
Normal	4	33.3
ALT		
Normal	8	66.7
High	4	33.3
Serum ammonia		
Normal	12	100
NPA test findings		
Adenovirus	1	8.3
Influenza A (H1N1)	6	50
Negative	5	41.7
**CSF glucose level mmol/L, mean (SD)**		4.2 (2.32)
**CSF protein level mmol/L, mean (SD)**		4.13 (10.27)
CSF culture		
Negative	10	83.3
Not done	2	16.7
Genetic study		
Negative	3	25
Not done	7	58.3
VUS	2	16.7

*ALT, Alanine aminotransferase; AST, Aspartate aminotransferase; CSF, Cerebrospinal fluid; NPA, Nasopharyngeal aspirate; VUS, variant of uncertain significance*.

### Genetic Testing

Genetic testing was done in five patients [whole exome sequencing (WES) in three and direct gene sequencing in two patients]. Genetic testing was not done for other patients either due to limited access to genetic testing or early deterioration and death in others. For the patients who had WES, the result was negative, while single gene sequencing for the *RANBP2* gene revealed a missense heterozygous variant in two patients [c.3363G>T, p. (Lys1121Asn) and c.128A>T, p. (Asp43Val)]. Both variants were classified as a variant of uncertain significance according to the recommendations of the American College of Medical Genetics (ACMG).

### Treatment and Outcomes

All cases were initially treated with antibiotics (mainly vancomycin and ceftriaxone) and antiviral (acyclovir) therapy. Intravenous methylprednisolone, 30 mg/kg daily for 3 days, and intravenous immunoglobulin (IVIG), 1 gm/kg daily for 2 days, was administered early in the course of the disease (within 72 h of presentation) in 10 cases (83.3%). The remaining two patients did not receive this regimen due to rapid deterioration and early death within the first 48 h. Tapering dose of oral steroids was used in six patients (50%). Plasma exchange (PLEX) was tried for two patients who did not show significant improvement. All patients underwent physiotherapy and speech therapy with variable responses.

Table 4 summarizes the treatment received. Outcomes were variable among our cases. Five patients had improvements in motor and cognitive functions; two patients had dystonia, two patients had speech delay, and early death in three patients (25%). Nine of the 12 patients had survived; all those survivors had received a combination of IVIG and IV methylprednisolone. Two patients who did not receive this combination of therapy died. However, statistical significance cannot be established due to singularity of the measured variables due to the small sample size. Table 5 shows prognoses and final outcomes. Survival was associated significantly with the use of IVIG and IV methylprednisolone combination (*P* = 0.043, Fisher's exact chi-squared test), demonstrated in Table 6.

**Table 4:** Treatments received.

	**Frequency (*n* = 12)**	**Percentage (%)**
Antibiotics (Vancomycin and Ceftriaxone combination)	12	100
Antiviral (Acyclovir)	12	100
Methylprednisolone	10	83.3
Tapering dose of oral Prednisolone	6	50
PLEX	2	16.7
Trial of Biotin and Thiamin	1	8.3
IVIG	10	83.3

*IVIG, intravenous immunoglobulin; PLEX, Plasma Exchange*.

**Table 5:** Prognosis and final outcomes: *n* = 12.

	**Frequency (*n* = 9)**	**Percentage (%)**
**Prognosis**		
Improved mental function	5	55.5
Improved motor function	5	55.5
Developed better proprioception	5	55.5
Speech delay problem	2	22.2
Moderate improvement (Mobility with independence)	4	44.5
Developed dystonia	2	22.2
**Final outcome**	**Frequency (*****n*** **=** **12)**	**Percentage (%)**
Survived	9	75
Deceased	3	25

**Table 6:** The Bivariate association between IVIG and IV methylprednisolone use with the survival: (*n* = 10).

	**Survival**		
	**Yes**	**No**	*p*-value^*^
Methylprednisolone +IVIG use			
No	0	2 (66.7)	0.045
Yes	9 (100)	1 (33.3)	

* *Fisher's exact chi-squared test of association*.

## Discussion

ANEC represents a fulminant type of encephalopathy which was initially described among Japanese and Taiwanese patients (2, 9). It is believed that ANEC has similar behavior to that of metabolic and immune-mediated disorders (10), but the exact pathophysiology is still unknown. It has multiple presumed causes that include previous infection, predominantly by influenza, herpes simplex virus, and human herpesvirus-6 (10–14). The affected children are more commonly to be those who are under 2 years of age. However, it can also affect older children as evidenced in this series. In the present study, all patients had a preceding viral illness, as evidenced by clinical presentation and virology screening.

Mizuguchi proposed specific criteria to establish the ANEC diagnosis (1). All patients had the proposed diagnostic criteria. Yagishita et al. (9) mentioned that it is not uncommon for patients with ANEC to have a normal CSF cell count. Similarly, all patients in our retrospective series had normal CSF findings. In accordance with previous studies, all patients showed bilateral thalamic lesions with variable involvement of other areas of the brain, including the basal ganglia, brainstem, cerebral white matter, and cerebellum. All of our patients had normal serum ammonia levels, which is one of the criteria proposed in the literature and noticed in previously reported cases (1, 9). Differential diagnoses, such as acute disseminated encephalomyelitis (ADEM) and other metabolic disorders like Leigh disease, organic acidemia, and Reye syndrome, need to be ruled out (15). The affected individuals with ANEC in this study were previously healthy, and basic metabolic studies were done with negative findings, including the absence of hypoglycemia, hyperammonia, and lactic acidosis as well as negative WES testing in three tested patients. In addition to the fact that previously reported cases did not have recurrent episodes (16). Reye syndrome can be differentiated from ANEC since the former is accompanied by hypoglycemia and hyperammonia and predominantly develops in patients treated with Aspirin (17).

Multiple studies have shown an association between *RANBP2* gene pathogenic variants and increased susceptibility to ANEC (6–8, 18–20). The *RANBP2* gene is located on chromosome 2q12.1-q13 and is part of the nuclear pore complex. This complex facilitates protein import and export and plays an important role in neuronal energy maintenance (20, 21). Heterozygous pathogenic variants in *RANBP2* gene and familial or recurrent ANEC have been reported in the literature, with the most common pathogenic variant being a missense, c.1754C>T, p.(Thr585Met). Those variants are inherited in an autosomal dominant fashion, and most of them have incomplete penetrance. On the other hand, there were cases in which no *RANBP2* variants were found, which suggests the existence of other predisposing factors (22, 23). In this study, both patients had *RANBP2* gene variants of uncertain significance according to the recommendations of the American College of Medical Genetics (ACMG).

The treatment of ANEC is not well-established and remains controversial. A previous study found that early intervention within 24 h of the presentation by IV Methylprednisolone 30 mg/kg/day for 5 days or dexamethasone 0.6 mg/kg/day divided into two to four doses for 2–4 days was associated with a good prognosis, while patients treated after 24 h had poorer outcomes. In the same study, patients who had brainstem lesions did not show significant recovery. On the other hand, IVIG administration was not shown to have significant prognostic value (24). Despite the prognostic value of administering IV Methylprednisolone within 24 h of presentation, the treatment of ANEC remains challenging as physicians hesitate to start their patients on IV Methylprednisolone early, since the condition may mimic herpetic encephalitis. The presence of characteristic neuroradiological features and initial negative CSF study may encourage treating physicians to consider the initiation of steroids early in the course of the disease. Nine of the patients in our study who received IV Methylprednisolone and IVIG had acceptable outcomes in motor and mental functions during their follow up. However, there were no standardized outcome measurements done for the patients. Survival was associated significantly with the use of IVIG and IV methylprednisolone combination. It is difficult to judge the efficacy of PLEX based on two patients. The prognosis of ANEC is generally variable; however, multiple studies have shown improvement over time (15). It is assumed that patients under 2 years old with increased liver enzyme levels and brainstem lesions have a poorer prognosis (9, 25–28). Liver function tests did not help determine vital prognosis. Liver enzyme levels were mildly to moderately increased in patients who died, while one case had a better prognosis despite the significantly higher aminotransferase levels. Brain edema and brainstem involvement, documented in five of our patients, led to variable outcomes, while the degree of severity of brainstem involvement may play an important role in determining prognosis.

### Limitations of the Study

This study has some limitations. First, it was a retrospective study, which did not allow us to determine a causal relationship between the tested parameters and the prognosis of ANEC, or to have standardized outcome measures. Second, the sample size was small, which limits the statistical analysis and generalization of results. Thirdly, we could not conduct functional studies on the found *RANBP2* variant, and there was no other data supporting pathogenicity.

## Conclusion

ANEC is a rare fulminant disease. Optimizing treatment tends to be challenging, but early recognition and intervention and early use of IVIG and IV Methylprednisolone may help improve outcomes. Further studies are needed to establish a consensus guideline for the management of such cases.

## Data Availability Statement

The data analyzed in this study is subject to the following licenses/restrictions: It is available if requested. Requests to access these datasets should be directed to Fahad A. Bashiri, fbashiri@ksu.edu.sa.

## Ethics Statement

The studies involving human participants were reviewed and approved by College of Medicine, King Saud University, Riyadh, Kingdom of Saudi Arabia (KSU-IRB Number 18/0683). Written informed consent from the participants' legal guardian/next of kin was not required to participate in this study in accordance with the national legislation and the institutional requirements.

## Author Contributions

All authors listed have made a substantial, direct and intellectual contribution to the work, and approved it for publication.

## Conflict of Interest

The authors declare that the research was conducted in the absence of any commercial or financial relationships that could be construed as a potential conflict of interest.
